# Cauterization of Cavity of the Uterus

**Published:** 1874-12

**Authors:** 


Cauterization of the Cavity of the Uterus.—Mr. Rawson, of Dublin, says, in the *Irish Hospital Gazette:* I use a very simple and handy instrument where I wish to cauterize the interior of the uterus. A silver catheter has the top taken off, and the edges carefully beveled slightly inward; the stilette is long enough to project about two inches; round the projecting part is carefully wrapped cotton wool, so that it can be pushed through the catheter and will not remain behind in the womb. Dr. Lombe Atthill has very clearly described how to do this (*vide* Braithwaite's Retrospect, vol. 69, p. 352). The patient lying on her back, I feel the os with the tip of my forefinger, and along this finger direct the catheter into the uterus, the cotton wool part of the stilette not reaching further than the eye of the instrument. On reaching the fundus, the stilette is pushed out while the catheter is withdrawn till it comes back to the os, when the stilette is withdrawn inside the catheter and the entire instrument removed. The cotton wool is saturated with whatever caustic I wish to use. This dispenses with the necessity of employing a speculum.

